# Validation of an automated dispensing system for subsequent dose dispensing of different radionuclides

**DOI:** 10.1186/s41181-023-00228-w

**Published:** 2024-02-12

**Authors:** T. T. Cao, E. A. Aalbersberg, M. M. Geluk-Jonker, J. J. M. A. Hendrikx

**Affiliations:** 1 Department of Nuclear Medicine, Antoni van Leeuwenhoek, Amsterdam, The Netherlands; 2 Department of Pharmacy & Pharmacology, Antoni van Leeuwenhoek Hospital, Plesmanlaan 121, 1066 CX Amsterdam, The Netherlands; 3 Present Address: GE Healthcare, Leiderdorp, The Netherlands; 4 Present Address: Cell Therapy Facility, University Medical Center Utrecht, Utrecht, The Netherlands

**Keywords:** Automated dispensing system, Radiopharmaceuticals, System validation, Performance qualification, GMP

## Abstract

**Background:**

Automated dispensing systems (ADSs) for radiopharmaceuticals have been developed to reduce the radiation exposure of personnel, to improve the accuracy of the dispensed dose and to limit the microbiological contamination. However, before implementing such systems, validation according to various applicable guidelines is necessary to ensure safety and quality. Here we present the selection, validation and implementation of the PT459R2 from manufacturer Lynax s.r.o. as a guidance protocol for validation according to GMP and GRPP guidelines. Validation included linearity accuracy and precision of the internal scintillation detector for different isotopes and microbiological validation for aseptic procedures.

**Results:**

The ADS can dispense accurate doses in the following linear range: 1000–10,000 MBq for lutetium-177, 20–74 MBq for zirconium-89, 100–1000 MBq for gallium-68 and 100–2000 MBq for fluorine-18. The maximum bias is 2.35% and the maximum coefficient of variation is 3.03% which meets the acceptance criteria of < 5%. Furthermore, the ADS does not affects the GMP class A environment in a laminar airflow cabinet and can dispense aseptically. In addition, radiation exposure is acceptable and data integrity is preserved.

**Conclusion:**

The PT459R2 ADS met all the requirements from our performance qualification and is therefore suitable for daily routine use in our center. Our approach can be used as a guidance for PQ of an ADS in a Radiopharmacy according to GMP and GRPP guidelines.

## Background

The use of radiopharmaceuticals for diagnosis and therapy has rapidly increased over the years. The compound growth rate for the radiopharmaceutical market is expected to be 8.2% annually in the period 2022–2028 (Zion Market Research 2023). This growth can lead to higher radiation exposure to the involved personnel who prepare the radiopharmaceuticals for administration. For this reason, automated dispensing systems (ADSs) have been developed. Literature has shown that using an automated dispensing system (ADS) can reduce the dose for the personnel substantially compared to manually dispensing a patient dose from a multi-dose vial (Covens et al. 2010). In addition, an ADS can improve the accuracy of the dispensed dose and limit the microbiological contamination compared to the standard procedure: manual preparation (Talbot et al. 2009; Martin et al. 2019). From the Good Manufacturing Practice (GMP) point of view, qualification of such systems is necessary to ensure that all stages are drawn up and tested in a controlled manner. The main stages of qualification in order include: user requirement specifications (URS), design qualification (DQ), factory acceptance testing (FAT), installation qualification (IQ), operational qualification (OQ) and performance qualification (PQ) (European Commission 2015; Todde et al. 2017). The URS defines the requirements for the use of the system that is in accordance with the applicable guidelines, laws and regulations and is used as a reference through most stages of the validation process and verifies whether the system complies to the established requirements and thus guidelines and regulations. Examples of these guidelines are the GMP guideline and the European Pharmacopoeia (Ph. Eur.), which both include requirements for (drug) substances, facilities and equipment to assure the quality of the pharmaceutical product (European Commission 2023; European Directorate for the Quality of Medicines HealthCare 2021). Moreover, systems used for radiopharmaceuticals require additional regulation and can sometimes seem contradictive as described by Lange (Lange et al. 2015). Firstly, there are the ICRP guidelines and Euratom Treaty, which are incorporated by individual countries into national legislation, for example into the Dutch Nuclear Energy Act in the Netherlands (ICRP 2007; Council Directive 2013; Nederlandse Overheid 1963). In addition to this, there are also European guidelines from the European Association of Nuclear Medicine (EANM) specific for Good Radiopharmacy Practice (GRPP) (Todde et al. 2017; Aerts et al. 2014; Gillings et al. 2020, 2021). In this article, a general validation strategy for an ADS for radiopharmaceuticals is reported while taking the various guidelines into account. This is the first report in literature on validation of a dispensing system for dispensing different radionuclides subsequently according to GMP and GRPP guidelines. In this study, we will follow the different validation stages for this ADS and investigate whether this system is suitable for application in our daily routine use.

## Methods

### Equipment

We used the PT314R2 ADS (LYNAX s.r.o., the Czech Republic) which was placed in a GMP grade A microbiological safety cabinet with a built-in movable lead glass for optimal shielding of the operator. The system can be loaded with a multi dose vial and is developed for dose dispensing of radiation-emitting radiopharmaceutical with the energy exceeding 400 keV. Multiple doses can be dispensed after each other by just replacing the syringe. By replacing the multi dose vial, different radiopharmaceuticals can be dispensed subsequently with very short time intervals in-between. In our center we intend to use it to dispense the following isotopes: Fluorine-18 (^18^F), Gallium-68 (^68^Ga), Zirconium-89 (^89^Zr) or Lutetium-177 (^177^Lu) products. For dose dispensing, different syringe sizes and matched syringe shields can be used. The system is controlled with the included Happy Brain software (version 4.4.36). The software is used to monitor, direct and control the dispensing system. In the software one can perform a daily stability test, determine the concentration of the placed vial and load (patient) data manually or by importing an Excel file. Furthermore, the historical data is automatically saved in the audit trail for traceability.

### Qualification stages

Equipment (including software) used in the production process of pharmaceuticals needs to be qualified and validated to ensure the quality of the product according to the GMP guidelines (European Commission 2015). The acceptance tests, IQ and OQ are often performed by the supplier and are therefore out of the scope of this manuscript. The PQ is carried out based on our established URS that focuses on the following points: (1) system validation, (2) aseptic production, (3) personnel safety, (4) data integrity.

#### System validation

For the system validation the linear range, accuracy and precision were investigated to determine whether the system can accurately dispense a dose within different dose ranges and volumina. Because the internal scintillation detector can measure within a broad energy range, multiple isotopes can be detected with their own efficiency curve each. Due to this, isotope specific validation is needed. In addition to this, each isotope has a different activity range for the daily use in the clinic. Based on the activity used in the clinic, a range for the validation was determined (see Table 1). To validate the linearity of the ADS in this range, five different doses for each isotope were dispensed in triplicate on three separate days. For Gallium-68 a sixth dose level was added since the dispensed volume in the lower range was expected to be limited. For each isotope, a multidose vial was prepared and placed in the ADS. For the validation of the linearity of dispensing, all doses were prepared in a 20 mL syringe.

**Table 1:** Measuring range of the automated dispensing system (ADS) for various nuclides

Nuclide	Accurate operational range as specified by manufacturer (MBq)	Range for validation based on locally used activity doses (MBq)	Nominal activity concentration in multidose vial
^177^Lu	100–10,000	1000–10,000	1000 MBq/mL
^89^Zr	0.2–50	10–74	10 MBq/mL
^68^Ga	10–2000	10–1000	100 MBq/mL
^18^F	10–3000	100–2000	185 MBq/mL

The acceptance criteria for the linearity are a correlation with a r^2^ > 0.99, a 95% confidence interval that contains 0 and a deviation in the dispensed activity of <  ± 5% from the calibration curve. The deviation between the requested dose and the acquired dose from the ADS was determined. For the accuracy and precision, a fixed activity per nuclide was dispensed in fivefold per syringe volume. Based on these results, the accuracy was determined as the bias and the precision as the coefficient of variation (CV) using the formulas 1 and 2. The accuracy and precision are accepted when the bias and CV are ≤  ± 10%.

1 $${\text{Bias }}\left[ \% \right] \, = \frac{{{1}00\% \, * \, ({\text{mean}}\;{\text{dispensed}}\;{\text{activity}}\;{\text{per}}\;{\text{ syringe }}{-}{\text{nominal}}\;{\text{activity}}) }}{{({\text{nominal}}\;{\text{activity}})}}$$

2 $${\text{CV}} \, [\% ] = \frac{{{1}00\% \, * \, ({\text{standard }}\;{\text{deviation}}\;{\text{ of}}\;{\text{dispensed}}\;{\text{activity}}\;{\text{per}}\;{\text{ run}})}}{{({\text{mean}}\;{\text{dispensed}}\;{\text{activity}}\;{\text{per }}\;{\text{syringe}})}}$$

#### Aseptic production

To determine the aseptic production with the ADS, an environmental and a production validation was executed. For the environmental validation, sedimentation and contact plates (Biotrading, The Netherlands) were used to determine the colony forming units (CFU) at high-risk spots during dispensing activities (in operation status). In addition, the Apex Z50 particle counter (Lighthouse, Unites States of America) was used to validate if the requirements for a GMP class A environment were preserved. For the production validation, the sterility was investigated by simulating the production using tryptic soy broth (Biotrading, The Netherlands). The broth did not contain radioactivity and was therefore dispensed by the ADS in the manual mode. Afterward the broth and the plates were incubated at 30–35 °C for 14 and 7 days respectively according to the European Pharmacopeia and Laboratory of Dutch Pharmacists (European Directorate for the Quality of Medicines & HealthCare 2023; LNA-procedures bereiding 2019, 2010). After incubation, the CFU on the plates were counted and further identification of colonies was done by an external ISO-15189 certified microbiology laboratory (Atal medial, the Netherlands) and in compliance with EU GMP chapter 7 (Outsourced Activities) (European Commission 2013). Concerning the broth, the turbidity was evaluated visually after 7 and 14 days.

#### Radiation protection

The theoretical dose rates during dose dispensing using the ADS have been determined prior to the purchase. During validation, the theoretical dose rates are compared to measurements in practice. The theoretical dose rates at 10 cm were calculated using an online calculator (Rad Pro Calculator, the United States of America). For the theoretical calculation an input of 0.4 mm tungsten shielding from the ADS was used. In addition, the dose rates were measured with a dose rate meter (Thermo Scientific FH-40G-L, the United States of America) at 10 cm from the vial placed in the ADS. Furthermore, we investigated if our requirements from the URS are accepted. Here we stated that the ADS must have sufficient shielding such that the maximum radiation exposure of 2 mSv/employee/year is not exceeded and the target is a maximum of 0.5 mSv/year if only one employee did all the work. For our center, employees are classified as radiation class B-workers where the annual dose limit is 6 mSv and the extremity limit is 150 mSv (Nederlandse Overheid 2023).

#### Data integrity

The GAMP5 provides a guideline for implementation and validation of automated systems and recommends that correctness of the transfer between two software systems is validated (International Society for Pharmaceutical Engineering 2022). The Happy Brain software cannot directly communicate with current used patient information software (IBC-NM, Comecer, Italy) despite having adequate and valid licenses for both software packages. However, an Excel export list from IBC-NM can be made and imported into the Happy Brain software. This eliminates the need to manually enter patient data from one system into the other with a greater risk of human error. For the validation, every time a dose was dispensed for e.g., the determination of the linearity, an Excel list was exported from IBC-NM and adjusted to the desired dosages. This list was then imported into the Happy Brain software and the syringes were dispensed based on this list. Afterwards an overview of the dispended doses was created via the Happy Brain software and compared to the originally exported list from IBC-NM.

## Results

### System validation

#### Linearity

**Fig. 1:**
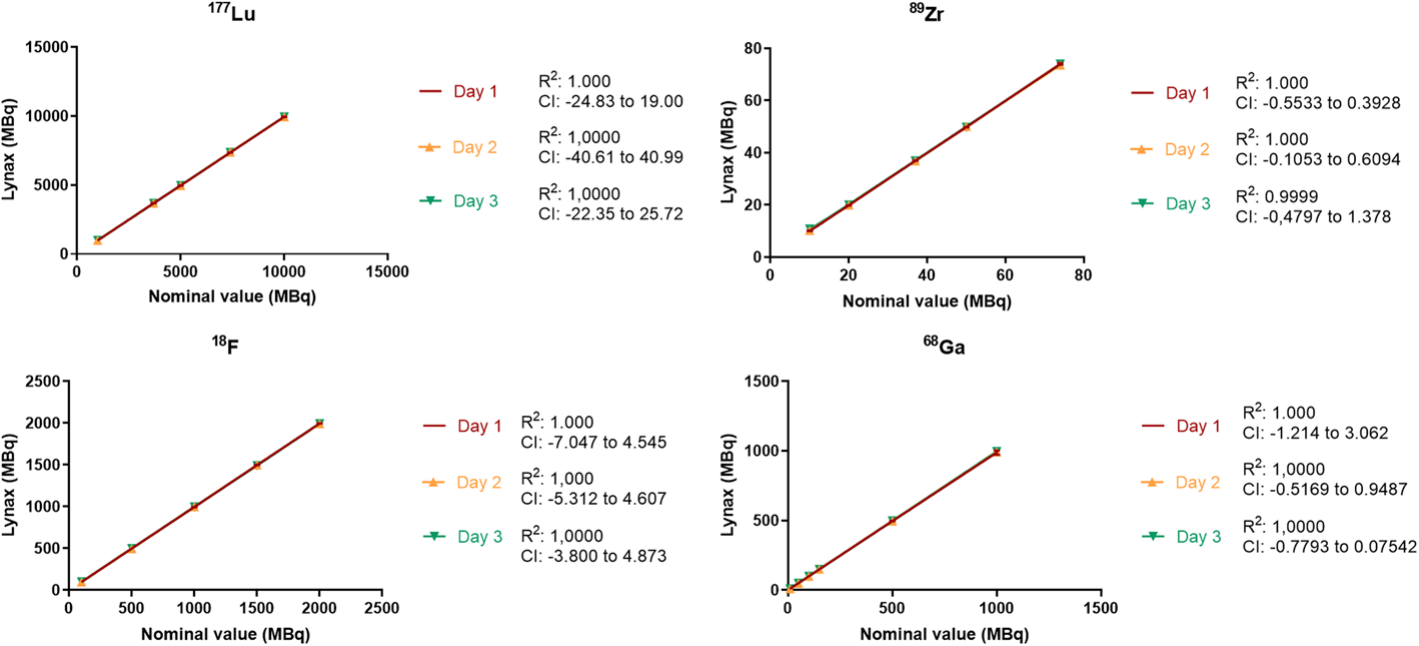
Linearity curves of dispensed doses. Curves show the dispensed dose (Y axe) versus the requested nominal dose per nuclide. On three different days, 3 syringes per dose were dispensed. Per day, a linearity curve was fitted for which the R^2^ and confidence intervals are shown

A simple linear regression fit was used for all nuclides (Fig. 1). Table 2 shows an overview of linearity for the four isotopes and the deviation at each measured concentration. For all nuclides, the deviation from the curve was < ± 5% for each dispensed dose. Only the mean deviation of dispensed doses of 10 MBq Zr-89 at day three was 7.42% (n = 3), resulting in a mean deviation of 3.57% over three days. Therefore, the validated linearity range for routine dose dispensing of Zr-89 products was set to 20–74 MBq, which is sufficient for a regular dose of 89-Zr products (usually 37 MBq). All the linearity curves had an r^2^ of > 0.99 and 95% confidence intervals that contained 0 and therefore met the acceptance criteria.

**Table 2:** Overview of the validated linearity parameters for each nuclide

Nuclide	^177^Lu	^89^Zr	^68^Ga	^18^F
Validated range (MBq)	1000–10,000	20–74	10–1000	100–2000
Mean deviation in dispensed dose over three days (n = 3 per day) per nomimal activity (Criteria: < ± 5%)	− 0.46% (10,000 MBq) − 0.29% (7400 MBq) − 0.50% (5000 MBq) − 0.34% (3700 MBq) − 0.76% (1000 MBq)	− 0.15% (74 MBq) − 0.27% (50 MBq) − 0.20% (37 MBq) − 0.30% (20 MBq) 3.57% (10 MBq)	− 0.70% (1000 MBq) − 0.50% (500 MBq) − 0.35% (150 MBq) − 0.52% (100 MBq) − 0.51% (50 MBq) − 1.15% (10 MBq)	− 0.26% (2000 MBq) − 0.24% (1500 MBq) − 0.36% (1000 MBq) − 0.28% (500 MBq) − 0.49% (100 MBq)
Mean r^2^ calibration curve (n = 3) (Criteria: > 0.990)	1.000	0.9999	1.000	1.000
95% confidence interval intercept (Criteria: interval contains zero)	Complies for each run (n = 3)	Complies for each run (n = 3)	Complies for each run (n = 3)	Complies for each run (n = 3)

#### Accuracy and precision

Table 3 shows an overview of the used parameters, bias and coefficient of variation. The intra-run accuracy of dispensed doses for all nuclides ranged between − 2.0 and 2.35% whereas the coefficient of variation ranged between − 0.59 and 1.70%. All the results met the acceptance criteria of a value < ± 5%.

**Table 3:** Overview of the bias and coefficient of variation for different syringe volumes

Nuclide	Syringe volume (mL)	Nominal activity (MBq)	Bias (%) (Criteria: < ± 5%)	Coefficient of variation (%) (Criteria: < ± 5%)
^177^Lu	30	100	0.77	1.70
	20	100	0.16	0.74
	3	40	− 2.00	3.03
^89^Zr	30	37	− 0.30	0.62
	20	37	− 0.38	0.39
	3	5	2.35	1.70
^68^ Ga	10	500	− 0.25	− 0.59
	5	300	− 0.19	0.36
	3	100	0.15	0.43
^18^F	10	1,000	− 0.51	0.19
	5	500	0.03	0.53
	3	100	− 0.17	0.47

### Aseptic production

The aseptic production validation consisted of three validation days, see Table 4 for an overview. For the environmental validation a total of fifteen settle plates were used during dispensing activities (in operation state) and one CFU was found after incubation. Determination showed that the bacteria was a gram-positive coccus which is a common bacterium in the microflora of our facility and commonly found on the skin and nasal mucosa of humans (Puligandla and Laberge 2006). The other parts of the environmental validation during dispensing activities met the requirements for a GMP class A environment. None of the nine contact plates showed growth of CFU and the particle count at rest (no working activities in the safety cabinet) and in operation (during dispensing activities) did not exceed the GMP limits. Furthermore, the production validation to validate whether the ADS can produce aseptically also passed the acceptance criteria. All the bouillons were clear and showed no growth of CFU’s.

**Table 4:** Overview of environmental monitoring after placing the automated dispensing system

	Day 1	Day 2	Day 3	Criteria GMP class A
Settle plates CFU/4 h average	0.2	0	0	< 1 CFU
Contact plates CFU/plate average	0	0	0	< 1 CFU
Particles at rest	0.5 µm: 24.7/m^3^ 5.0 µm: 0.0/m^3^	0.5 µm: 42.4/m^3^ 5.0 µm: 0.0/m^3^	0.5 µm: 219.1/m^3^ 5.0 µm: 7.1/m^3^	0.5 µm: ≤ 3520 /m^3^ 5.0 µm: ≤ 20/m^3^
Particles in operation	0.5 µm: 38.9/m^3^ 5.0 µm: 0.0/m^3^	0.5 µm: 7.1/m^3^ 5.0 µm: 0.0/m^3^	0.5 µm: 732.9/m^3^ 5.0 µm: 12.0/m^3^	0.5 µm: ≤ 3520 /m^3^ 5.0 µm: ≤ 20/m^3^
Bouillon simulation	Clear medium	Clear medium	Clear medium	Clear medium

*CFU* colony forming units

### Radiation protection

The measured and theoretical dose rates at 10 with shielding can be found in Table 5. The shielding used at 10 cm was 0.4 mm tungsten provided by the ADS. The measured dose rate for most isotopes was lower than the calculated dose rates and therefore met the acceptance criteria of shielding sufficiently. Only the measured dose for ^177^Lu was higher than the theoretical dose. The difference was 4.2 uSv/h and therefore can be explained by an increased background radiation by other products in the environment. Furthermore, the calculator had no option for Ga-68. Based on the properties of Ga-68 as a PET isotope, a similar dose rate to F-18 is expected and therefore within the acceptance criteria.

**Table 5:** Overview of the dose rates for the different nuclides in their most common dose

Nuclide (activity)	Measured Dose rate at 10 cm with 0.4 mm tungsten shielding (µSv/h)	Theoretical dose rate at 10 cm with 0.4 mm tungsten shielding (µSv/h)
Lu-177 (7400 MBq)	13.5	9.3
Zr-89 (37 MBq)	150	393
Ga-68 (150 MBq)	300	–
F-18 (300 MBq)	1200	1902

### Data integrity

The dispensed syringes for the system validation (180 syringes) were all dispensed from an adapted export list from our current patient information system. A total of 12 export lists were created and imported into the Happy Brain software. Of all these lists, there was a 100% correct transfer between the two different operating systems.

## Discussion

This manuscript describes an example of a performance qualification for an automated dispensing system for subsequent dose dispensing of multiple radionuclides. In literature, guidelines and guidance on the validation of automated systems in radiopharmacy are available (Aerts et al. 2014; Gillings et al. 2021; Todde et al. 2017). However, the content is mainly focused on the IQ and OQ, which are often performed by the supplier. The PQ in contrast is often only briefly described and indicates that one should be perform a PQ based on a risk assessment of the specific premise. In this manuscript, we provide an applicable PQ of an ADS for radiopharmaceuticals based on our URS that is in line with the GMP requirements.

During validation, deviations from the PQ were necessary for the accuracy and precision measurements. We initially wanted to dispense a fixed dose of 100 MBq for all the isotopes and syringe volumes. However, in practice it was not always possible to make several dilutions due to the short half-lives of the PET isotopes. Furthermore, when using a 20 or 30 mL syringe, the volume of a 100 MBq dose was lower than the specified minimum volume for dose dispensing in 20 or 30 mL syringes. This altogether made that we dispensed the most suitable dosage at that time regarding the concentration. The chosen values therefore may appear random but ultimately the goal was to demonstrate that the ADS can dispense accurately and precise within a range of used syringe volumes. This has been demonstrated in this PQ and emphasizes the flexibility needed with radiopharmaceuticals.

Relevant literature was used as a backbone for our validation. It was found that other articles on the validation of an ADS often only describe one specific topic. For example, one article focuses on the accuracy and precision of an ADS compared to the convention manual method (Nazififard et al. 2013). Another topic that is often highlighted when validating an ADS, is the (extremity) dose reduction, which has been described for [^18^F]FDG (Covens et al. 2010; O’Doherty et al. 2014). More recently, a more complete validation of an ADS with injector system which also includes the sterility was reported. That ADS (MEDRAD, United States of America), however, is used as a stand-alone dose dispenser and injector and placed in a clinical patient setting whereas our ADS is placed in a Class II safety cabinet with laminar airflow (down flow), placed in a cleanroom of a radiopharmacy production facility and has to comply with the pharmaceutical regulatory framework (Lecchi et al. 2012). In our PQ we have included all the above-mentioned topics. Here it is important to not only focus on the working of the system but also whether it is safe to use, both for personnel and ultimately for patients.

In our PQ, an additional environmental validation in accordance to GMP annex 1 (version of 25 November 2008) was executed. However, recently a new version of annex 1 was published (version of 2 August 2022, coming into operation on 25 August 2023). The main differences involving our manuscript are that the maximum limits for total particle ≥ 5 µm/m^3^ is set from 20 to not specified and that the maximum permitted CFU for a GMP class A is set from < 1 CFU to no growth. In our worst-case operational environmental validation, growth of one CFU was found and according to GMP, action should be taken. For this we determined the CFU, investigated where it is commonly found and how to prevent it. In addition, all the other plates were clean and therefore we can conclude that the ADS does not affect the GMP classification. Furthermore, a daily ongoing process validation regarding aseptic production is performed to see monitor the trend (Martin et al. 2019).

## Conclusion

We described a practical guidance for the PQ of an ADS according to GMP and GRRP guidelines using the validation of the PT314R2 ADS as an example. Determination of PQ requirements based on an URS and the subsequent validation were described. The validation results met all the requirements from our performance qualification for dispensing of individual syringes of Fluorine-18, Gallium-68, Zirconium-89 and Lutetium-177 containing radiopharmaceuticals from a multi-dose vial. The ADS is linear in the ranges applicable for our daily clinical use; has an accuracy and precision that complies to the acceptance criteria; does not affect the GMP classification; can produce aseptically; have acceptable radiation exposure and data integrity. In conclusion, our ADS is suitable for the aseptic dispensing of individual patient syringes from a multidose vial with fast switching between different radionuclides and is therefore applicable for our daily routine use. Our approach can be used as a guidance for PQ of an ADS in a Radiopharmacy according to GMP and GRPP guidelines.

## Data Availability

The datasets used and/or analysed during the current study are available from the corresponding author on reasonable request.

## Abbreviations

ADS: Automated dispensing system
CFU: Colony forming units
DQ: Design qualification
EANM: European Association of Nuclear Medicine
FAT: Factory acceptance testing
GMP: Good manufacturing practice
GRPP: Good Radiopharmacy Practice
IQ: Installation qualification
OQ: Operational qualification
Ph. Eur: European Pharmacopoeia
PQ: Performance qualification
URS: User requirement specification
